# How Stress Is Related to Age, Education, Physical Activity, Body Mass Index, and Body Fat Percentage in Adult Polish Men?

**DOI:** 10.3390/ijerph191912149

**Published:** 2022-09-25

**Authors:** Monika Lopuszanska-Dawid, Przemysław Kupis, Anna Lipowicz, Halina Kołodziej, Alicja Szklarska

**Affiliations:** 1 Department of Human Biology, Faculty of Physical Education, Józef Piłsudski University of Physical Education in Warsaw, 00-968 Warsaw, Poland; 2 Department of Anthropology, Faculty of Biology and Animal Science, Wroclaw University of Environmental and Life Sciences, 50-375 Wroclaw, Poland; 3 Polish Academy of Sciences, Poland, Palace of Culture and Science, Defilad Square 1, 00-901 Warsaw, Poland

**Keywords:** Wroclaw Male Study, stressor, educational level, health behaviors, Poland

## Abstract

Stressful events and chronic tension are considered a burden and a threat to physical, mental, and social health. The aim of the study was to demonstrate the associations of variation in stress exposure with social factors, physical activity, basic components of physical fitness, body mass index (BMI) and percentage of body fat (BFP). An additional objective was to identify the main BFP modifiers among those analyzed. The material consisted of data of ethnically homogeneous group 355 men (32–87 yrs), invited to the study as part of the Wroclaw Male Study research project. The analyzed features included socioeconomic status (age, educational level), elements of lifestyle (physical activity), major and most important stressful life events—Social Readjustment Rating Scale (SRRS) and basic parameters of the somatic structure of the body (BMI, BFP). Statistical analyses included: chi-square test, Mann–Whitney U test and backward stepwise regression (significance level α = 0.05). Stress exposure showed significant socioeconomic variation among the adult Poles studied. Higher levels of education were associated with higher levels of stress. Significant correlations between SRRS and physical activity were found, especially in men older than 60 years and with higher levels of education. A positive relationship was shown between SRRS and BFP, especially in men under 60 years of age. BFP appeared to depend mainly on age and stress. The main determinants of SRRS were age and education level, while BFP turned out to be more sensitive to stress than BMI. The modifying force of physical activity for SRRS appears to be age dependent.

## 1. Introduction

In most societies, stressful events and chronic tension are recognized as a burden and threat to both physical, mental, and social health, as has been repeatedly demonstrated empirically [1,2,3,4]. The more stressful and negative events a person encounters, the more likely they are to suffer injury, illness, disability, or even death [5,6]. Accumulating misfortune, worry, and affliction necessitate many life changes over a short period of time, often exceeding a person’s ability to cope and adapt, increasing vulnerability to infection, injury, and illness. Consequently, emotional disorders, inflammation, migraine, muscle pain, skin diseases, respiratory disorders, gastric disorders, and additionally other serious diseases such as obesity, autoimmune diseases, depression, and atherosclerosis may develop [7,8,9,10]. Not insignificant are the consequences in social health, which can manifest as conflicts with others, exclusion, and problems with building relationships [2]. Many studies dealing with stress in the context of health prevention mainly consider significant life events, often unexpected [11]. Other researchers indicate that it is chronic tensions and ongoing difficulties, although of lower magnitude and expected, that may have worse health effects. Examples of such tensions include family conflicts, difficult financial situation, disability coexisting with dependence on others, problems at work, and unfavorable environments [8,11]. It is suggested that these ongoing difficulties may have a stronger impact on mental health than single and more severe events, such as wars or natural disasters [12,13,14,15]. When analyzed together, unexpected stressors along with chronic tensions and ongoing difficulties can explain up to 40% of the variation in psychological distress and depressive symptoms [12,13].

Although being increasingly studied, the health consequences of stress still require in-depth and multifaceted analyses to identify the most important modifiers [16]. In most cases, stress is triggered by the occurrence of a stressor that threatens or limits control and causes negative emotions and agitation. The duration of stress-induced reactions and their intensity are determined by several factors, mainly related to the characteristics of a given stressor and the possibilities of recovery from the situation [17]. Many coping strategies are known, among them the three main ones: being task-focused, emotion-focused, and avoidance-focused. From the perspective of this study, and because the data does not include task-focused measures, avoidance-focused and emotion-focused coping appear to be of key importance. In many cases, using avoidance may, unfortunately, have worse health consequences than the stressor itself [18]. Dealing with stress aimed at regulating emotions, mainly lowering unpleasant emotional states and strengthening positive emotions, will rather have a positive effect on health, mainly on the immune and endocrine system [19]. There are also some moderators that mitigate these reactions and their effects, including support from others, individual psychophysical endurance, self-esteem, coping skills, preparation for circumstances, and exercise [14].

Among the adult populations of many countries, one of the key modifiers of the intensity of perceived stress is the social position associated with individuals’ educational levels and occupational status. Some researchers point to the varying abilities to adapt to stressors of both individuals and entire social groups, meaning that the effects of long-term psychosocial stress may vary socially. The observed variation in sensitivity to stress stimuli, but also unequal stress coping abilities, may determine the overall body response or group reaction. In this light, differences in the level of experienced psychosocial stress are considered to be one of the important reasons for the presence of social gradients in biological measures of health [20].

Stress-induced health effects can occur both directly, through specific bodily responses to stressors that occur [21], and indirectly, through changes in health behavior, habits, or decisions that may be risky to health. Unhealthy diet, overweight, physical inactivity, smoking tobacco, alcohol abuse, and irregular sleep are examples of changes in basic lifestyle elements that undoubtedly have implications for human health [17,22]. Unhealthy behavior is seen as rewarding and soothing in the context of stress [23]. Associations between stress and these variables have been repeatedly demonstrated in research, although analyzing them is not easy because of the multiplicity of other relevant factors, and the findings are not always in agreement [17]. The impact of stress on health behavior can vary in direction and strength, which depend on many factors (mainly personal) and the stage of life a person is at. Lack of sense of control, low self-esteem, and stressor-induced anxiety can foster unhealthy behavior (if not going to include dispositional factor). Health behavior is particularly important from the perspective of individual biological condition, as it becomes habitual and has a significant impact on health condition [24]. It is usually considered a way to cope with stress. The likelihood of falling into unhealthy habits under stress has been shown to be higher in young people, even before working age. Adults, on the other hand, are more likely to change health behavior than youth. The nature of the effect of such a change that occurs under stress is age-dependent. Another equally important concept is the reverse causality of stress and health behavior. Engaging in certain health behaviors may increase or decrease the likelihood of a stressor [24].

Research has shown that lifestyle modifications to stressors are highly dependent on psychological predispositions and can be dichotomously different, such as increasing the habit of snacking versus lack of appetite and aversion to food intake [25], increasing alcohol consumption versus abstinence, or increasing versus decreasing tobacco smoking [23,26,27]. Stress-induced eating behavior varies according to gender, baseline body mass index, and other individual preferences and factors [28]. One such factor is cortisol, which, when secreted as a response to stress, has a direct effect on weight gain. Cortisol leads to fat accumulation and inhibits growth hormone secretion, which would counteract this accumulation. Therefore, exposure to the effects of stress through elevated cortisol levels can lead to fat accumulation. However, the production of this hormone has wide individual variation [29]. Many studies have explored the importance of stress as a factor in weight gain and future obesity through eating disorders associated with physical inactivity [28]. However, the relationships shown are often weak or not always present, which may be due to the time it takes to gain or lose weight from the time the stressor occurred, so the effects are observed with some delay, even several months. The body mass index, or BMI, which has been most commonly chosen for testing, is also a significant limitation, and in many cases is subject to a significant error [30].

The aim of the study was to demonstrate the directions and strength of stress exposure variability with social factors, physical activity, basic physical fitness factors, body mass index (BMI), and percentage of body fat (BFP). The necessity to carry out in-depth research in this area results from the existing gap in the scientific literature. Most of the research concerns single-factor relationships of the stress-BMI type. The presented results focus on multifaceted and multilevel relationships, including groups of modifiers of perceived stress, such as social factors, lifestyle elements and basic parameters of biological condition. In today’s turbulent times, the issue of stress and its effect on an individual’s life requires in-depth and multifaceted research. Further exploration of modifiers of perceived stress and its health effects appears to be particularly important during unstable and stressful periods. Identification of groups at risk of elevated stress may have important practical implications, especially in terms of education on methods of coping with stressors to mitigate its health consequences.

## 2. Materials and Methods

### 2.1. Sample

The material for the study consisted of sociological and biomedical data of 355 men aged 32–87 years, randomly invited to the study as part of the Wroclaw Male Study research project (WMS) [20,31,32,33,34,35]. The men were active, healthy inhabitants of the city of Wroclaw (Poland, Lower Silesia, population in 2010: 632,996, including men: 294,960), and they were medically examined in 2000 and in 2010/11 under the WMS research project in the Lower Silesian Medical Centre DOLMED S.A. in Wroclaw (Poland).

The group has been described in detail in previous publications, especially the most recent one [20]. The group was ethnically homogeneous (Poles, Caucasian) with no national, linguistic, religious, or racial minorities. This population had no history of any physical and mental disorders. On physical examination, they presented no pathologies. Men included in the study did not suffer from any essential chronic diseases actually and had not done so in the past. Subjects taking medications, especially drugs that could interfere with hormone metabolism or mental health, were excluded from the analysis. The men were not selected for any biological parameters or in another way. To eliminate the impact of human mobility on the biological condition, the important inclusion criteria were that participants of this study were inhabitants of the city of Wroclaw [36]. The original group consisted of 384 individuals, but 7.6 percent of men were excluded from the analysis and the final database for further analysis included 355 men. The enrolment rate was similar to that observed in Poland and amounted to about 25 percent [37,38]. Unfortunately, determining the direction of possible material selectivity is impossible [20]. It is probable that only individuals with a high level of health awareness and volunteers for all types of preventive medical examinations took part in this research project. Thus, the results obtained may be more favorable than expected.

### 2.2. Ethical Approval

The study proposal was approved by the Bioethical Committee of Medical University in Wroclaw, Poland (approval number: 477/2000) and conducted in accordance with the Helsinki Declaration and its later amendments or comparable ethical standards. All persons were orally informed about the aims of the projects and all testing procedures and all gave their informed consent prior to their inclusion in the study. At any time, the subjects could withdraw without giving any reason. All participants signed informed consent.

### 2.3. Measurements

#### 2.3.1. Qualitative Survey Data

The respondents completed a questionnaire containing basic information about their socioeconomic status: age (in years) (1: under 50 yrs, 2: 50–59 yrs, 3: 60–69 yrs, 4: 70 yrs and more), educational level (1: university, 2: secondary school, 3: vocational (and/or primary school)), and level of physical activity (1: inactive, 2: medium, 3: active).

#### 2.3.2. Anthropometric Measurements

The relative body mass index (BMI) was calculated based on self-measured body height and body weight according to the standard Martin technique [39,40,41]. Body height was measured as the distance of the vertex point from the basis (B-v: Basis–vertex) while maintaining the Frankfurt plane by the Martin anthropometer (GMP Gneupel Präzisions-Mechanik, Zurich, Switzerland) with an accuracy of 1 mm [42]. Although it is more time-consuming, body height measurements give much more reliable results than in the case of body height declared by study participants [43]. Body weight (to the nearest 0.1 kg) and percentage of body fat (PBF) (to the nearest 0.1%) were measured during the same examination, using a scale with a Soehnle Professional 7850 body composition analyzer (Soehnle Industrial Solutions GmbH, Backnang, Germany). The measurements were taken in the morning. The participants were standing upright, without shoes, in their underwear, and they were fasting. For participants up to age 65, normal BMI was adopted as 18.5–24.9 [44], whereas for age 65 and over, this was 25.0 to 27.0 [45,46]. There were no subjects with a below-normal BMI among the participants.

#### 2.3.3. The Social Readjustment Rating Scale

The Social Readjustment Rating Scale (SRRS) [47] was used for identifying major and most important stressful life events. SRRS helps assess the stress load, more specifically, objective stressors, i.e., those that cause characteristic symptoms in all or almost all people. The tool is formed by a list of 43 stressful life events that are assigned corresponding Life Change Units (LCU) on a scale of 0 to 100, depending on how traumatic an impact they had in the study on the project participants (from *Death of spouse* rated as 100 LCU to *Change in eating habits* rated as 15 LCU) [20]. Stress is cumulative; in order to estimate the experienced total stress, the scores from each event that occurred over a 12-month period must be added together (which gives the total number of stressful life events for an individual). The authors noted a significant positive relationship between the magnitude of stress and health, and thus, as the number of LCUs increases, so does the incidence of diseases, i.e., the risk of stress-related health breakdown. There is a statistically significant correlation between the strength of stressors and the probability of developing a serious illness within two consecutive years. If the total strength of stressors is 150–199 LCU points, the probability of getting sick is 37%, 200–299 LCU points: 51%, over 300 LCU points: 79% [47]. Because the sample under study consists of a substantially healthy group and due to the small number of respondents declaring stressor levels 150 LCU points and above, the original scale has been extended and three additional lowest levels of strength of perceived stressors have been introduced: 0 LCU points, 1–49 LCU points, 50–149 LCU points. Details: In this study, the SRRS scale modified by the authors was used. The following categories of LCU stress points were used: 1: 0 points, 2: 1–49 points, 3: 50–149 points, 4: 150–199 points, 5: 200–299 points, 6: over 300 points. Categories 1–3 were introduced by the authors to refine the original category 0–149 points. In order to estimate the amount of the probability of developing a serious illness within two consecutive years, the authors calculated that since the original 0–149 points category generates max. 36% risk, therefore 1 point generates 0.24% risk (36%/149 points = 0.24% per points). Therefore the levels of strength of perceived stressors were distinguished 1–6, which may correspond to, respectively, approx. 0%, 6%, 24%, 37%, 51% and 79% of the risk. Method for calculating the risk for the selected new categories (1–3): 1: 0 points—0 * 0.24% = 0% of the risk; 2: 1–49 points—((1 * 0.24%) + (49 * 0.24%))/2 = 12%/2 = 6%; 3: 50–149 points—((50 * 0.24%) + (149 * 0.24%))/2 = 47.76%/2 = 23.88%, therefore rounded to the whole—24%.

### 2.4. Statistical Analysis

Descriptive and mathematical statistical methods were used to present the results (e.g., medians, percentage frequencies). The distributions of continuous variables were not normal, as verified using the Shapiro–Wilk and Jarque–Bera tests and therefore non-parametric tests were applied: chi-square test (χ^2^) and Mann–Whitney U test. Differences between the proportions of age and educational level categories (respectively: under 50 yrs, 50–59 yrs, 60–69 yrs, 70 yrs and more; university, secondary, vocational school) in terms of level of physical activity (inactive, medium, active), BMI (in the norm, above of the norm), BFP categories (10–19%, 20–29%, 30%+) and stress categories (0, 1–49, 50–149, 150 and more points) were analysed with the use of the chi-square test. The Mann–Whitney U test was used to evaluate the mean differences between the independent samples (respectively: 1. in terms of age and education levels, 2. levels of physical activity in terms of age and educational level, 3. BMI categories in age and education groups, 4. PBF categories in groups age and educational level).

The main modifiers of the basic physical fitness parameter of the male participants were selected in the course of the subsequent analysis steps in the manuscript and confirmed in the literature. Backward stepwise regression was used to assess the strength of the relationships of the main modifiers of the basic biological characteristics of the studied men. BFP was a dependent variable, whereas the age, stress (as continuous variables), educational level and physical activity (as qualitative variables) were independent variables (not continuous variables were used as a dummy variables). The basic model containing all potential independent variables was constructed, and then the variables were gradually eliminated from the model to maintain the final model with the highest value of the coefficient of determination while retaining only significant parameters. The values of standardized regression coefficient (β—Beta), level of statistical significance (*p*—*p*-value), R-squared values of determination (R^2^), the adjusted R^2^ values of determination (R^2^ adj.) (information about the percentage of the variance of the dependent variable explained by the determining variable) and model adjustment Fisher test (F) were given. The level of significance was set at α = 0.05. The STATISTICA 13.5, and 12.0. packages were used for analyses (Tulusa, OK, USA) [48].

## 3. Results

Table 1 part A shows the prevalence of males in the stress categories, followed by age and education categories and the differences in distribution. There were statistically significant differences in distribution of stress intensity with age and education (respectively, *p* = 0.003 and *p* = 0.008). Higher levels of stress (150 points and more) are more common in younger age groups (25.8% among men under 50 yrs), while the 60 and over group reported the lowest stress exposure (60–69 yrs: 5.5%, 70 yrs and more: 11.8%). College education, compared to vocational or high school education, had different distributions of the proportions of stress categories. People with higher levels of education were more likely to report higher categories of stress exposure (university versus vocational school—1–49 LCU points: 29.6% versus 17.5%; 50–149 LCU points: 32.1% versus 28.6%; 150+ LCU points: 17.0% versus 6.3%).

Table 1 part B, illustrating the results of the Mann–Whitney U test, reveals significant differences (*p* = 0.001) in median stress between categories under 50 years and 60–69 years and between 50–59 years and 60–69 years (model I). The median stress of the participants aged 60–69 yrs and 70 yrs and more was found to be lower, but not statistically significant, than the median for those under 60 (respectively, Me = 23 and Me = 39 versus Me = 40 for men under 50 yrs and Me = 51 for 50–59 years old men). In the model II, two age groups were compared: under 60 yrs and 60 years old and over (younger versus older). The median stress of the participants aged under 60 yrs was found to be significantly higher than the median for those under 60 (respectively, Me = 49.5 versus Me = 25). For education, a significant difference occurred between vocational and high school education and between vocational and college education (respectively, *p* = 0.001, *p* = 0.028). The median stress of those with vocational education was the lowest (Me = 12) (Table 1, part B).

The above results formed the basis for assumptions in further analyses. Hence, the subsequent analyses compare two age groups: under 60 and 60 years old and over (younger versus older) and two educational groups: college and high school education combined and vocational education (higher levels of education versus lower levels of education). This approach is supported by the lack of the statistically significant differences between the median stress for men under 50 and 50–59 years of age, and for men aged 60–69 and 70 and more (Table 1, part B, model I), the occurrence of significant statistical differences between the median stress for men under 60 and 60 yrs and more (Table 1, part B, model II), and the biological differences in men up to 60 and after 60 years of age identified in previous scientific studies concerning Polish men [30].

Table 2, part A shows the prevalence of males in the stress categories, followed by physical activity level by two age groups and two education groups and the differences in distribution. There were statistically significant differences in distribution of stress intensity with physical activity for men aged 60 yrs and more (*p* = 0.011) and education for men with higher levels of education (*p* = 0.002). Among men 60 yrs and more, higher levels of stress are more common in active men (LCU = 150+: 7.8% among active versus 3.7% among inactive; LCU = 50–149: 36.7% among active men versus 29.6% among inactive). Most of the Polish men (60 yrs and more) with lower stress levels reported the lowest physical activity (LCU = 0: 59.3% inactive men versus 25.6% active). Among men with higher levels of education, inactive compared to medium or active had different distributions of the proportions of stress categories. Physically active, well-educated men were more likely to report higher categories of stress exposure (active versus inactive–1–49 LCU points: 31.2% versus 11.1%; 50–149 LCU points: 32.4% versus 25.9%; 150+ LCU points: 17.3% versus 16.7%).

The results of the Mann–Whitney U tests (Table 2 part B) indicated that statistically significant differences for median stress were obtained in the 60+ group between inactive and active individuals (*p* = 0.008) and between moderately active and active individuals (*p* = 0.030). In this group, the median stress of the very active individuals (Me = 39) was found to be significantly higher than for other groups (inactive and moderately active, Me = 0 and Me = 5.5, respectively). This result confirms and clarifies the results presented above. For the more highly educated group, there was a significant difference between the inactive and the active individuals (*p* = 0.040). The median stress of very active (Me = 49) versus inactive individuals (Me = 21.5) was more than twice as high.

Table 3, part A presents the prevalence of males in the stress categories, followed by Body Mass Index by two age groups and two education groups and the differences in distribution. There were no statistically significant differences in distribution of stress intensity with BMI categories for age and education categories (respectively, *p* = 0.437 for men under 60 yrs; *p* = 0.611 for men 60 yrs and more; *p* = 0.715 for well-educated; *p* = 0.639 for poorly educated). However, a trend can be observed that, among men the highest level of stress, the normal range of BMI was more common (for example, in the group under 60 yrs with LCU = 150+ 23.3% men had BMI in the norm versus 17.9% were above of the norm; in group 60 yrs and more, respectively: 8.6% versus 7.1%) (Table 3, part A).

Table 3, part B, illustrating the results of the Mann–Whitney U test, reveals no significant differences in median stress between Body Mass Index categories under age and education groups (*p*-values range between 0.078 and 0.788).

Table 4, part A presents the prevalence of males in the stress categories, followed by Body Fat Percentage categories by two age groups and two education groups and the differences in distribution. Although the results of the χ^2^ were found to be statistically insignificant (*p*-values range between 0.111 and 0.517), it is worth noting that, in the group with lower stress categories (e.g., LCU = 0), men with the highest category of BFP (30% and more) were slightly more frequent (among men under 60 yrs: 35.5% had BFP 30%+ versus 16.7% had BFP 10–19%; among men 60 yrs and more: respectively 44.4% versus 20.0%). There were no statistically significant differences in distribution of stress intensity with BFP categories for education categories, but the trends in the proportions are similar to those described slightly above. Among men from the lowest stress category (LCU = 0), a slightly larger fraction of men had BFP above 30% than BFP within 10–19% (in the group with higher levels of education had LCU = 0, 36.6% and BFP = 30% and more, whereas 18.8% had 10–19%; in groups with lower levels of education, the corresponding values are 60.9% versus 0%).

The results of the Mann–Whitney U tests (Table 4 part B) indicated that statistically significant differences for median stress were obtained in the younger group (under 60 yrs) between men with a BFP of 10–19% and over 30% (*p* = 0.012). The median stress in men with more than 30% (Me = 25) body fat percentage appeared to be significantly lower than in those with the lowest body fat (Me = 88). In the group with a university and secondary level of education, the Mann–Whitney U test scores were significantly different between those with 10–19% and over 30% (*p* = 0.005) and between 20–29% and over 30% body fat (*p* = 0.017). The median stress (LCU points) for groups 10–19%, 20–29% and over 30% were, respectively, 52, 48 and 32). For the group with vocational education, a significant difference also occurred between those with 10–19% and over 30% body fat (*p* = 0.027), with an equally significant difference in medians of Me = 65 to Me = 0, respectively.

Given the significance of the associations of stress exposure with age, education, physical activity level, and body fat in a backward regression model, we examined the extent to which BFP, being one of the main parameters of biological fitness, depends on a set of other factors. Table 5 presents the results of backward stepwise regression (both basic and final models), with age, education, physical activity level, and stress being the independent variables and BFP being the dependent variable.

The basic model fit R^2^ was 21.30, which means that the independent variables (age, education, physical activity, SRRS) explain more than 21% of the variation in body fat of the male participants. At a significance level of 5%, age and SRRS were found to significantly affect the percentage of body fat. For age, a positive relationship with SRRS and a β coefficient of 0.413 were found, whereas, for SRRS, the relation was negative at a coefficient of −0.102. Subsequent backward regression steps discarded irrelevant variables (educational level and physical activity level), and as a result, the model adjustment increased from 23.408 to 45.099. The final model indicates that body fat percentage, with other factors controlled, increases with age and decreases with level of SRRS exposure.

## 4. Discussion

Stress exposure among male respondents is noticeably socially differentiated. There was also a significant positive correlation between SRRS and physical activity, particularly pronounced for men over 60 years and with a high school or college education. Furthermore, a significant positive relationship was found between SRRS and body fat for men under 60 years of age and in all education groups. It is difficult to unequivocally indicate the direction of the relationship between SRRS and physical activity and body fat, but it appears that education largely determines the results obtained for these variables.

### 4.1. Stress and Education

A social diversity analysis that took into account the age and education of the participants led to predictable conclusions, which were consistent with those of other authors. Older people appeared to be less stressed. The limit of change in the proportional distribution of SRRS categories was around 60 years of age. Other studies have found that stress levels peak in early adulthood [24]. Stressors such as success in the job market, getting married, and raising children are observed in early or middle adulthood and begin to fade over time. In contrast, the decrease in stress at later ages may be related to the fact that people are transitioning or approaching retirement, thus becoming less exposed to work-related stress. The reduced number of commitments, stressful situations, and a kind of quietude associated with the transition into old age may explain the reduced exposure to stress during this period of life [49]. For the group analyzed in the study, the decline in stress intensity with age may be even stronger due to the high proportion of college-educated individuals whose overall life situation, e.g., financial and health status, may be better than that of their less-educated peers.

Types of stressors, whether related to the socioeconomic environment or the nature of the work performed, tend to be passed from generation to generation, gradually increasing the differences in exposure between advantaged and disadvantaged groups [2]. Stress exposure is unevenly distributed in the population and can cause disparities in well-being, and mental and physical health. Exposure to stressors, according to some studies, is inversely related to social status, putting those with lower social status at a greater disadvantage. They have to face more stress and overcome more tensions [7,11]. This disparity in exposure to stressful events and coping skills may explain health disparities between different social groups [50]. Difficulties and stress occurring in one domain of life can also easily spread to other domains, e.g., from work to home, and cause problems to accumulate [51,52].

The effect of stressors on health and general well-being may be weaker for individuals who have high self-esteem and support from others, use appropriate coping strategies and experience, and are prepared for the stressors that occur [2]. These social and individual assets can effectively undermine the effects of stressors and prevent the process of their accumulation and reproduction. However, these assets are unevenly distributed among social groups, leading to the widening of already existing disparities [2]. A perfect example of this is men and women who approach coping with stressful situations differently by choosing different strategies. Men tend to focus on task-oriented strategies, while women often choose emotion-oriented strategies and seek social support [53].

The results of the present study concerning education indicate that men who were least stressed were the least educated, followed by those with vocational and high school education. The explanation of this can be also found in the literature, suggesting a link between higher levels of education and more demanding occupations. Having such jobs entails higher responsibility, pressures, and more potential stressors, which may be reflected in the individual responses of study participants [17]. However, most scientific papers and the arguments posed in them point to an inverse relationship between stress and education than that obtained here. It is suggested that better-educated people use better coping strategies, have higher self-esteem, better support from those around them, and have greater overall control over their lives [54,55]. It is difficult to argue with such reasoning, but one should take into consideration the strength of the factors mentioned. However, it is worth referring again to the specifics of the group studied, since the age of the participants may be a period in which work is the main and strongest source of stress, overshadowing other factors, even those that are supposed to have a protective effect.

### 4.2. Stress and Physical Activity

In the context of stress, physical activity is often described as a sort of buffer to mitigate the effects caused by the stressor. Physically active leisure activities serve to calm and relax all body systems. Those who choose this strategy report lower levels of stress than their peers who do not. However, this assessment is subjective and does not necessarily correspond to the actual level of stress exposure [56]. In addition to physical activity, studies have also analyzed physical fitness, which in itself, as physical resistance to a stressor, or indirectly through psychological benefits (e.g., higher self-esteem, endurance) [57], may facilitate coping with stress. It is difficult to separate the effects of these two variables (physical activity and physical fitness) as they are correlated to some extent. As research shows, both can lead to physical and psychological symptoms caused by stress. The intensity and type of physical activity turn out to be of little importance compared to the amount of time spent on it. Active leisure becomes an effective escape from stressor-induced pressures and a process that adds new energy and vigor to face stress. In this context, regular participation in physical activity becomes a kind of protective activity because stress, especially increased stress, is inherently unpredictable [14]. The literature also indicates that highly stressed individuals tend to choose passive leisure activities because, unlike physical activity, they are immediately rewarded with rest and passivity [23]. The results obtained in this study concerning PA seem to confirm mainly the latter direction of the relationship. Physically inactive respondents were significantly less stressed than very active respondents, whether broken down by age group or education group. Highly stressed respondents were more likely to be physically active, using physical activity to relieve the effects (tension) caused by stress.

### 4.3. SRRS, BMI, and BFP

Basic biological status parameters such as BMI and percentage of body fat showed significantly different and distinct associations with SRRS. BFP was found to be highly significantly associated with stress while BMI was not at all. The men most exposed to stressful events were found to have the lowest levels of body fat, while the least exposed men had the highest levels. In the context of the positive associations of higher levels of physical activity in men with higher stress exposure, the results for BFP are consistent, although different from what is typically presented in research [28]. It seems that, again, education and age may explain such findings. Analysis of other variables would require operating on age-education subgroups, which is not possible due to the sample size because education and age may determine exposure to stressors much more strongly than other variables analyzed in this study. A recent analysis of the determinants of body fat showed that, even with other variables controlled, a significant negative relationship between stress and body fat is observed. This fact also suggests the previously neglected but arguably fundamental problem of endogeneity i.e., cause-and-effect feedback, which we cannot resolve due to a lack of access to relevant instrumental variables.

### 4.4. Limitation Study

When proceeding to any interpretation of the results obtained in this study, one should take into account a relatively specific study group consisting of relatively well-educated men living in large cities, and the specificity of the stress testing tool. Future research devoted to this topic would benefit from combining both the subjects’ filling the SRRS questionnaire and a question of their subjective perceptions of stress. The comparison of such results could allow for a deeper analysis of the problems studied and a search for explanations and cause-and-effect relationships. Additionally, the use of a modified by authors version of the SRRS scale may be a certain limitation of the study, although it seems that extending the scale may enrich the inference. Subsequent limitation studies can be used a subjective questionnaire to assess the level of physical activity, instead of other objective methods, e.g., accelerometers. Thus, when generalizing the results to the general population, the limitation study must be considered.

## 5. Conclusions

The issue of stress and its importance to an individual’s life still seems to be neglected these days. Education in methods of coping with stressors and the health effects of exposure to prolonged or intense stress is either absent or occurs superficially without practical value. It seems particularly justified to educate adolescents and young adults in this field, since the accumulation of stress in early adulthood should be preceded by adequate preparation for this period of life. Further scientific exploration of these issues is not without significance, as many questions remain without reliable and proven answers.

## Figures and Tables

**Table 1:** Associations of stress with age and education: Part A—number of observations (n) and proportions (%) of stress categories by age and education groups and results of χ^2^ and *p*; Part B—results of the Mann–Whitney U test for stress by age and education groups.

**A part**	Stress Categories (LCU)								Sum	
	0		1–49		50–149		150+			
	n (%)		n (%)		n (%)		n (%)		N (%)	
Age (years)										
under 50 yrs	20 (22.5)		25 (28.1)		21 (23.6)		23 (25.8)		89 (100)	
50–59 yrs	25 (23.8)		27 (25.7)		39 (37.1)		14 (13.3)		105 (100)	
60–69 yrs	43 (39.1)		26 (23.6)		35 (31.8)		6 (5.5)		110 (100)	
70 yrs and more	18 (35.3)		12 (23.5)		15 (29.4)		6 (11.8)		51 (100)	
All ages	106 (29.9)		90 (25.4)		110 (31.0)		49 (13.8)		355 (100)	
χ^2^, *p*	25.011, 0.003									
Educational level										
university	34 (21.4)		47 (29.6)		51 (32.1)		27 (17.0)		159 (100)	
secondary school	42 (31.6)		32 (24.1)		41 (30.8)		18 (13.5)		133 (100)	
vocational school	30 (47.6)		11 (17.5)		18 (28.6)		4 (6.3)		63 (100)	
All	106 (29.9)		90 (25.4)		110 (31.0)		49 (13.8)		355 (100)	
χ^2^, *p*	17.244, 0.008									
**B part**	n	Me (LCU)	U	*p*	U	*p*	U	*p*	U	*p*
Age (years, model I)			under 50 yrs		50–59 yrs		60–69 yrs		70 yrs+	
under 50 yrs	89	40	-	-	-	-	-	-	-	-
50–59 yrs	105	51	0.735	0.462	-	-	-	-	-	-
60–69 yrs	110	23	3.399	0.001	3.210	0.001	-	-	-	-
70 yrs and more	51	39	1.522	0.128	1.098	0.272	−1.287	0.198	-	-
Age (years, model II)			under 60 yrs				60 yrs nad more			
under 60 yrs	194	49.5	-		-		-		-	
60 yrs and more	161	25	3.608		0.001		-		-	
Educational level			university		secondary school		vocational school			
university	159	48	-	-	-	-	-		-	
secondary school	133	39	−1.369	0.171	-	-	-		-	
vocational school	63	12	−3.403	0.001	−2.192	0.028	-		-	

Legend: Stress Categories (LCU)—stress categories are cumulative LCU (Life Change Units) of stressful life events for the preceding 12-months; n—sample size with specific parameters; N—total sample size; yrs—years; χ^2^—chi-square test, *p*—*p*-value, level of significance level; Me—median; U—Mann–Whitney U test.

**Table 2:** Associations of level of physical education and stress (with age and education control): Part A—number of observations (*n*) and proportions (%) of stress categories by age and physical activity level and results of χ^2^ and *p*; Part B—results of the Mann–Whitney U test for stress by age and physical activity level.

**A part**	Stress Categories (LCU)						Sum	
Physical activity	0	1–49	50–149		150+			
	n (%)	n (%)	n (%)		n (%)		N (%)	
Age: under 60 yrs								
inactive	13 (35.1)	4 (10.8)	10 (27.0)		10 (27.0)		37 (100)	
medium	10 (21.7)	14 (30.4)	18 (39.1)		4 (8.7)		46 (100)	
active	22 (19.8)	34 (30.6)	32 (28.8)		23 (20.7)		111 (100)	
Sum	45 (23.2)	52 (26.8)	60 (30.9)		37 (19.1)		194 (100)	
χ^2^, *p*	12.548, 0.051							
Age: 60 yrs and more								
inactive	16 (59.3)	2 (7.4)	8 (29.6)		1 (3.7)		27 (100)	
medium	22 (50.0)	9 (20.5)	9 (20.5)		4 (9.1)		44 (100)	
active	23 (25.6)	27 (30.0)	33 (36.7)		7 (7.8)		90 (100)	
Sum	61 (37.9)	38 (23.6)	50 (31.1)		12 (7.5)		161 (100)	
χ^2^, *p*	16.512, 0.011							
Education: higher levels of education								
inactive	25 (46.3)	6 (11.1)	14 (25.9)		9 (16.7)		54 (100)	
medium	18 (27.7)	19 (29.2)	22 (33.8)		6 (9.2)		65 (100)	
active	33 (19.1)	54 (31.2)	56 (32.4)		30 (17.3)		173 (100)	
Sum	76 (26.0)	79 (27.1)	92 (31.5)		45 (15.4)		292 (100)	
χ^2^, *p*	20.871, 0.002							
Education: lower levels of education								
inactive	4 (40.0)	0 (0.0)	4 (40.0)		2 (20.0)		10 (100)	
medium	14 (56.0)	4 (16.0)	5 (20.0)		2 (8.0)		25 (100)	
active	12 (42.9)	7 (25.0)	9 (32.1)		0 (0.0)		28 (100)	
Sum	30 (47.6)	11 (17.5)	18 (28.6)		4 (6.3)		63 (100)	
χ^2^, *p*	9.357, 0.154							
**B part**	n	Me (LCU)	U	*p*	U	*p*	U	*p*
Age: under 60 yrs			inactive		medium		active	
inactive	37	74	-	-	-	-	-	-
medium	46	42	0.505	0.614	-	-	-	-
active	111	48	0.085	0.933	−0.500	0.617	-	-
Age: 60 yrs and more							-	-
inactive	27	0	-	-	-	-	-	-
medium	44	5.5	−0.637	0.524	-	-	-	-
active	90	39	−2.657	0.008	−2.171	0.030	-	-
Education: higher levels of education								
inactive	54	21.5	-	-	-	-	-	-
medium	65	40	−0.725	0.469	-	-	-	-
active	173	49	−2.054	0.040	−1.458	0.145	-	-
Education: lower levels of education								
inactive	10	63.5	-	-	-	-	-	-
medium	25	0	0.943	0.346	-	-	-	-
active	28	12	1.241	0.215	−0.152	0.879	-	-

Legend: Stress Categories (LCU)—stress categories are cumulative LCU (Life Change Units) of stressful life events for the preceding 12-months; n—sample size with specific parameters; N—total sample size; yrs—years; χ^2^—chi-square test; *p*—*p*-value, level of significance level; Me—median; U—Mann–Whitney U test.

**Table 3:** Associations of stress with age and BMI category: Part A—number of observations (n) and proportions (%) of stress categories by age and BMI category and results of χ^2^ and *p*; Part B—results of the Mann–Whitney U test for stress by age and BMI category.

**A part**	Stress Categories (LCU)					Sum
Body Mass Index	0	1–49	50–149	150+		
	n (%)	n (%)	n (%)	n (%)		N (%)
Age: under 60 yrs						
In the norm	9 (20.9)	8 (18.6)	16 (37.2)	10 (23.3)		43 (100)
Above of the norm	36 (23.8)	44 (29.1)	44 (29.1)	27 (17.9)		151 (100)
Sum	45 (23.2)	52 (26.8)	60 (30.9)	37 (19.1)		194 (100)
χ^2^, *p*	2.720, 0.437					
Age: 60 yrs and more						
In the norm	11 (31.4)	11 (31.4)	10 (28.6)	3 (8.6)		35 (100)
Above of the norm	50 (39.7)	27 (21.4)	40 (31.7)	9 (7.1)		126 (100)
Sum	61 (37.9)	38 (23.6)	50 (31.1)	12 (7.5)		161 (100)
χ^2^, *p*	1.817, 0.611					
Education: well-educated						
In the norm	15 (22.1)	18 (26.5)	22 (32.4)	13 (19.1)		68 (100)
Above of the norm	61 (27.2)	61 (27.2)	70 (31.3)	32 (14.3)		224 (100)
Sum	76 (26.0)	79 (27.1)	92 (31.5)	45 (15.4)		229 (100)
χ^2^, *p*	1.358, 0.715					
Education: poorly educated						
In the norm	5 (50.0)	1 (10.0)	4 (40.0)	0 (0.0)		10 (100)
Above of the norm	25 (47.2)	10 (18.9)	14 (26.4)	4 (7.5)		53 (100)
Sum	30 (47.6)	11 (17.5)	18 (28.6)	4 (6.3)		63 (100)
χ^2^, *p*	1.691, 0.639					
**B part**	n	Me (LCU)	U	*p*	U	p
Age: under 60 yrs			In the norm		Above of the norm	
In the norm	43	90				
Above of the norm	151	40	1.763	0.078		
Age: 60 yrs and more						
In the norm	35	39				
Above of the norm	126	23	0.790	0.429		
Education: higher levels of education						
In the norm	68	12.5				
Above of the norm	224	39	1.650	0.099		
Education: lower levels of education						
In the norm	10	8				
Above of the norm	53	12	0.269	0.788		

Legend: Stress Categories (LCU)—stress categories are cumulative LCU (Life Change Units) of stressful life events for the preceding 12-months; n—sample size with specific parameters; N—total sample size; yrs—years; χ^2^—chi-square test; *p*—*p*-value, level of significance level; Me—median; U—Mann–Whitney U test.

**Table 4:** Associations of stress with age and BFP category: Part A—number of observations (n) and proportions (%) of stress categories by age and Body Fat Percent category and results of χ^2^ and *p*; Part B—results of the Mann–Whitney U test for stress by age and BFP category.

**A part**	Stress Categories (LCU)						Sum	
Body Fat Percentage	0	1–49	50–149		150+			
	n (%)	n (%)	n (%)		n (%)		N (%)	
Age: under 60 yrs								
10–19%	8 (16.7)	13 (27.1)	16 (33.3)		11 (22.9)		48 (100)	
20–29%	26 (22.6)	30 (26.1)	36 (31.3)		23 (20.0)		115 (100)	
30%+	11 (35.5)	9 (29.0)	8 (25.8)		3 (9.7)		31 (100)	
Sum	45 (23.2)	52 (26.8)	60 (30.9)		37 (19.1)		194 (100)	
χ^2^, *p*	5.215, 0.517							
Age: 60 yrs and more								
10–19%	1 (20.0)	3 (60.0)	1 (20.0)		0 (0.0)		5 (100)	
20–29%	28 (33.3)	22 (26.2)	26 (31.0)		8 (9.5)		84 (100)	
30%+	32 (44.4)	18 (18.1)	23 (31.9)		4 (5.6)		72 (100)	
Sum	61 (37.9)	38 (23.6)	50 (31.1)		12 (7.5)		161 (100)	
χ^2^, *p*	7.102, 0.312							
Education: higher levels of education								
10–19%	9 (18.8)	14 (29.2)	14 (29.2)		11 (22.9)		48 (100)	
20–29%	38 (23.2)	45 (27.4)	53 (32.3)		28 (17.1)		164 (100)	
30%+	29 (36.3)	20 (25.0)	25 (31.3)		6 (7.5)		80 (100)	
Sum	76 (26.0)	79 (27.1)	92 (31.5)		45 (15.4)		292 (100)	
χ^2^, *p*	10.333, 0.111							
Education: lower levels of education								
10–19%	0 (0)	2 (40.0)	3 (60.0)		0 (0)		5 (100)	
20–29%	16 (45.7)	7 (20.0)	9 (25.7)		3 (8.6)		35 (100)	
30%+	14 (60.9)	2 (8.7)	6 (26.1)		1 (4.3)		23 (100)	
Sum	30 (47.6)	11 (17.5)	18 (28.6)		4 (6.3)		63 (100)	
χ^2^, *p*	8.465, 0.206							
**B part**	n	Me (LCU)	U	*p*	U	*p*	U	*p*
Age: under 60 yrs			10–19%		20–29%		30%+	
10–19%	48	88						
20–29%	115	53	1.352	0.177				
30%+	31	25	2.528	0.012	1.703	0.089		
Age: 60 yrs and more								
10–19%	5	36						
20–29%	84	38	−0.290	0.772				
30%+	72	21.5	0.075	0.940	1.126	0.260		
Education: higher levels of education								
10–19%	48	52						
20–29%	164	48	1.174	0.240				
30%+	80	32	2.811	0.005	2.393	0.017		
Education: lower levels of education								
10–19%	5	65						
20–29%	35	12	1.924	0.054				
30%+	23	0	2.212	0.027	0.728	0.467		

Legend: Stress Categories (LCU)—stress categories are cumulative LCU (Life Change Units) of stressful life events for the preceding 12-months; n—sample size with specific parameters; N—total sample size; yrs—years; χ^2^—chi-square test; *p*—*p*-value, level of significance level; Me—median; U—Mann–Whitney U test.

**Table 5:** Results of backward stepwise regression (basic and final models)—BFP as the determined variable, age, educational level, physical activity and SRRS as the determining variables.

	Basic Model		Final Model	
	β	*p*	β	*p*
age	0.413	0.001	0.421	0.001
educational level	0.076	0.118	-	-
physical activity	0.043	0.363	-	-
SRRS	−0.102	0.036	−0.110	0.024
R^2^	21.30		20.58	
Adj. R^2^	20.39		20.13	
F (*p*) (model adjustment)	23.408 (*p* < 0.001)		45.099 (*p* < 0.001)	

Legend: β—Beta standardized regression coefficient; F—Ronald A. Fisher’s test; *p*—*p*-value, level of significance level; R—R-values of determination; R^2^—R-squared values of determination; R^2^ adj.-the adjusted R-squared values of determination.

## Data Availability

All relevant data are within the manuscript. The selected data underlying the results represented in the study are available from the authors. There are ethical/legal restrictions on sharing a raw data set, which includes many confidential data (sensitive patient information) (EU Regulation EU, 2016/679 of the European Parliament and of the Council of 27 April 2016 on the protection of natural persons with regard to the processing of personal data and on the free movement of such data, and repealing Directive 95/46/EC—General Data Protection Regulation, https://eur-lex.europa.eu/eli/reg/2016/679/oj, accessed on 1 January 2022). If necessary, access will be given.
